# A Case Report of Acute Infective Endocarditis Caused by Aggregatibacter aphrophilus Involving the Tricuspid Valve

**DOI:** 10.7759/cureus.64412

**Published:** 2024-07-12

**Authors:** Ana Jesus, Manuela Lopes, Paula Martins, António Pires

**Affiliations:** 1 Pediatric Cardiology, Unidade Local de Saúde de Coimbra, Hospital Pediátrico, Coimbra, PRT; 2 Pediatric Cardiology, Faculdade de Medicina da Universidade de Coimbra, Coimbra, PRT

**Keywords:** pulmonary embolization, aggregatibacter aphrophilus, hacek group, tricuspid valve, infective endocarditis

## Abstract

We report a case of a 16-year-old male with tricuspid valve infective endocarditis caused by *Aggregatibacter aphrophilus* and complicated by pulmonary septic embolisms. Multiple antimicrobial therapy was unsuccessful and surgical management was required. In this report, the authors highlight the importance of a high index of suspicion regarding the diagnosis of endocarditis and its possible complications.

## Introduction

*Aggregatibacter aphrophilus* is part of the HACEK group of organisms (*Haemophilus *spp., *Aggregatibacter actinomycetemcomitans*, *Cardiobacterium hominis*, *Eikenella corrodens*, and *Kingella kingae*), which are fastidious gram-negative bacteria that typically inhabit the normal microbiota of the oral and upper respiratory tract in humans [1]. Despite its generally low pathogenicity, *A. aphrophilus* is implicated in approximately 1-3% of all cases of infective endocarditis (IE) [1]. Involvement of the tricuspid valve is rare and is typically associated with risk factors such as congenital heart disease, intravenous drug use, cardiac devices, or the presence of indwelling vascular lines [2-4].

## Case presentation

**Figure 1:**
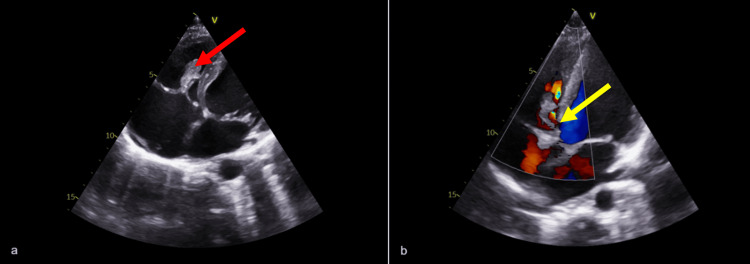
Four-chamber transthoracic echocardiogram (a) Large vegetation adherent to the tricuspid septal leaflet (red arrow) measuring 23 x 9 mm; (b) Left-to-right shunt through a small perimembranous ventricular septal defect (VSD) (yellow arrow).

We report a case of a 16-year-old male who presented to the emergency room with a one-week history of chest pain, cough, fatigue, and high fever (peaking at 40 degrees Celsius). The patient resided in a childcare institution and his past medical history included a perimembranous restrictive ventricular septal defect (VSD) and a recent bout of gingivitis. Four days prior to admission, he was diagnosed with a self-limiting upper respiratory tract infection. On examination, he was sweaty and had a grade III/VI holosystolic heart murmur, best audible in the lower left sternal border. The chest X-ray was normal, and analytically he had microcytic hypochromic anemia (8.3 mg/dL), leukocytosis (31,600 cells/uL) with a high neutrophil count (28,540 cells/uL), and a C-reactive protein of 12 mg/dL. Respiratory virus screening was negative, and blood samples were taken for culture and serological testing. Transthoracic echocardiography confirmed the presence of a VSD and revealed a moderate tricuspid insufficiency, as well as tricuspid septal leaflet vegetation measuring 23 × 9 mm (see Figure 1).

Due to the high suspicion of IE, he was empirically started on intravenous ampicillin, flucloxacillin, and gentamicin, as per the 2023 European Society of Cardiology (ESC) Guidelines [5]. Blood culture was positive for *A. aphrophilus*, and antibiotic therapy was changed to ceftriaxone 2 grams intravenously twice daily. Despite adequate antimicrobial management, the pyrexia persisted (maximum of 40 degrees Celsius) every four to six hours, and the size of the vegetation remained unchanged.

**Figure 2:**
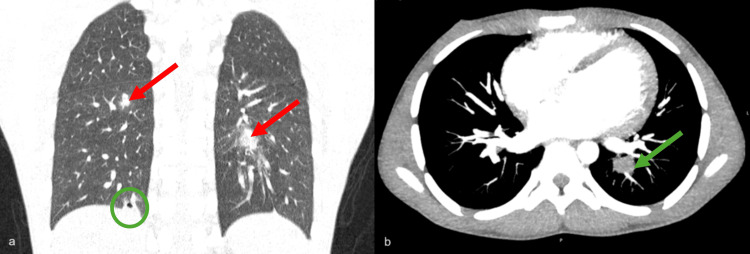
Thoracic CT scan (a) Coronal axis - nodular images consistent with septic emboli in the left mediobasal segment and in the upper segment of the right lower lobe (red arrows). Also visible is a cavitated nodule in the right posterobasal segment (green circle); (b) Axial axis - a septic embolus, measuring 15 mm, is seen in the left mediobasal segment, accompanied by endoluminal filling of two segmental arterial branches (green arrow).

On day 10 post-admission, due to continued chest pain and persistent fever, a thoracic computed tomography angiogram was carried out which showed several pulmonic septic emboli (see Figure 2). Ciprofloxacin was added to the antibiotic regimen, but no clinical improvement was observed. Due to failed medical therapy, on day 17 post-admission, he underwent surgical intervention, with direct closure of the VSD, removal of the tricuspid septal leaf vegetation, and a De Vega-modified tricuspid annuloplasty. The postoperative period was uneventful and the antibiotic therapy was adjusted to ceftriaxone 2 grams intravenously twice daily, according to the agent's susceptibility. The fever subsided 13 days post-surgery, with the patient remaining clinically stable. Subsequently, he was discharged upon completion of a six-week course of intravenous antibiotics, according to the 2023 ESC Guidelines [5].

## Discussion

IE is an inflammation of the inner lining of the heart chambers and heart valves, caused by a bacterial or, less commonly, a fungal infection [5]. Its diagnosis remains a significant challenge, with notable mortality rates persisting to this day [6,7]. Right-sided IE accounts for only 5% to 10% of all patients, with the tricuspid valve being more commonly affected than the pulmonary valve. IE diagnosis is often difficult, and a high suspicion index is needed. It should be considered in patients presenting with prolonged fever of unknown origin, especially when associated with risk factors such as congenital heart disease, valvular heart disease, recent dental or surgical procedures, or immunosuppression [5]. Transthoracic echocardiography is key to diagnosis, providing crucial information regarding the presence of vegetation, its characteristics, size, and potential local complications [5,8]. Blood cultures are also essential for both diagnosis and susceptibility testing, although isolating certain microorganisms can pose some challenges. *A. aphrophilus*, a gram-negative bacteria belonging to the HACEK group, is commonly associated with dental infections, as it is part of the normal oropharyngeal flora [1]. Its fastidious growth nature renders it difficult to isolate in blood cultures, and suspicion should arise in cases of suspected IE cases with negative blood cultures. Polymerase chain reaction (PCR) testing for this agent may be useful in culture-negative endocarditis. It is usually susceptible to cephalosporins and has a good prognosis when treated timeously and adequately. The recommended duration of treatment is usually four to six weeks [5]. Embolic complications are not uncommon, with the brain and spleen being primary sites for embolisms in left-sided cases [1], whereas pulmonary embolisms are more common in cases of right-sided IE and IE involving pacemaker leads. Several factors are associated with an increased risk of embolism including the size, mobility, and location of the vegetation, particular microorganisms, previous embolism, and multivalvular involvement [5,9]. Its presence should be suspected in cases of persistent fever [10]. Surgery may play a role in the treatment of infections unresponsive to antibiotic therapy [5].

The lack of antibiotic prophylaxis for IE in patients with perimembranous VSDs remains a clinical dilemma. Although the American Heart Association (AHA) does not recommend IE prophylaxis for patients with VSDs, considering them low risk compared to other cardiac defects [11], the risk of IE in patients with unrepaired VSDs can be approximately 30 times higher than in the general population [12-14]. Despite this high risk, the ESC guidelines still recommend surgical VSD closure only for patients with repeated episodes of IE [5]. The National Institute for Health and Clinical Excellence (NICE) working group has previously determined there is insufficient evidence to justify the use of prophylactic antibiotics to reduce the incidence of IE during dental procedures. However, this stance has been questioned, especially considering the morbidity associated with IE when it occurs post-dental work. Evidence has suggested that prophylactic antibiotics can reduce bacteremia levels following dental procedures, potentially lowering the risk of IE [15]. It may be prudent to revisit the guidelines and practices concerning antibiotic prophylaxis, especially for this group of patients.

## Conclusions

We present a case of tricuspid valve IE on a 16-year-old with a history of a VSD and gingivitis, that was unresponsive to intravenous antibiotics and needed surgical treatment. Congenital heart disease and dental issues should alert clinicians of the possibility of endocarditis in children and teenagers with prolonged high fevers of unknown origin. Tricuspid valve endocarditis is uncommon and embolic complications should be suspected in cases of no response to appropriate antibiotic treatment. While cases of *A. aphrophilus* are scarce in the literature, they appear to carry a favorable prognosis.
